# Modified Alphavirus-Vesiculovirus Hybrid Vaccine Vectors for Homologous Prime-Boost Immunotherapy of Chronic Hepatitis B

**DOI:** 10.3390/vaccines8020279

**Published:** 2020-06-05

**Authors:** Carolina Chiale, Timur O. Yarovinsky, Stephen W. Mason, Bhaskara R. Madina, Manisha Menon, Marie M. Krady, Safiehkhatoon Moshkani, Anasuya Chattopadhyay Pal, Bijan Almassian, John K. Rose, Michael D. Robek, Valerian Nakaar

**Affiliations:** 1 Department of Immunology and Microbial Disease, Albany Medical College, Albany, NY 12208, USA; chialec@amc.edu (C.C.); moshkas@amc.edu (S.M.); robekm@amc.edu (M.D.R.); 2 Department of Pathology, Yale University School of Medicine, New Haven, CT 06510, USA; anasuya.chattopadhyay@yale.edu (A.C.P.); john.rose@yale.edu (J.K.R.); 3 CaroGen Corporation, Farmington, CT 06032, USA; smason@carogencorp.com (S.W.M.); bmadina@carogencorp.com (B.R.M.); manisha.menon9@gmail.com (M.M.); mkrady@carogencorp.com (M.M.K.); balmassian@carogencorp.com (B.A.)

**Keywords:** hepatitis B virus, vesicular stomatitis virus, RNA replicon, immunotherapy

## Abstract

Virus-like vesicles (VLV) are hybrid vectors based on an evolved Semliki Forest virus (SFV) RNA replicon and the envelope glycoprotein (G) from vesicular stomatitis virus (VSV). Previously, we showed that VLV can be used to express protein antigens and generate protective antigen-specific CD8^+^ T cells. This report describes VLV vectors designed for enhanced protein expression and immunogenicity. Expressing hepatitis B virus (HBV) middle S antigen (MHBs) from VLV using a dual subgenomic promoter significantly increased MHBs-specific CD8^+^ T cell and antibody production in mice. Furthermore, envelope glycoprotein switch from VSV Indiana to the glycoprotein of Chandipura virus enabled prime-boost immunization and further increased responses to MHBs. Therapeutic efficacy was evaluated in a mouse model of chronic HBV infection initiated by HBV delivery with adeno-associated virus. Mice with lower or intermediate HBV antigen levels demonstrated a significant and sustained reduction of HBV replication following VLV prime-boost immunization. However, mice with higher HBV antigen levels showed no changes in HBV replication, emphasizing the importance of HBV antigenemia for implementing immunotherapies. This report highlights the potential of VLV dual promoter vectors to induce effective antigen-specific immune responses and informs the further development and evaluation of hybrid viral vaccine platforms for preventative and therapeutic purposes.

## 1. Introduction

VLV are a capsid-free, self-replicating virus-like vaccine platform carrying positive-strand capped and polyadenylated RNA encoding an in vitro evolved SFV RNA-dependent RNA replicase and the VSV glycoprotein [1]. VLV can be engineered to express foreign antigens and induce robust immune responses [2,3]. The VLV platform replicates like a virus, but its only structural protein is the VSV glycoprotein (VSV-G), and unlike many other viral vectors, it lacks pathogenicity [4,5]. In infected cells, the newly translated SFV RNA replicase synthesizes complementary negative-strand RNA, full-length positive-strand RNA, and a smaller mRNA encoding VSV-G [2,6]. The VLV vector was further evolved through 50 passages in baby hamster kidney (BHK)-21 cells to produce high titers in cell culture [7]. In previous studies, we showed immunogenicity of VLV expressing antigens from hepatitis B virus (HBV) in mice and human immunodeficiency virus (HIV) in non-human primates [4,8].

Stable expression of foreign antigens by the evolved VLV was achieved through the insertion of an RNA sequence encoding the picornavirus T2A ribosome skipping site upstream of VSV-G [8,9,10]. However, the T2A site adds extra amino acids to the C-terminal domain of the protein [9], which may negatively impact protein expression, stability, or secretion, and reduce antibody responses to those antigens [8,11]. Our previous studies with HBV showed that expression of MHBs alone or in combination with other HBV antigens resulted in MHBs-specific T cell activation [8,10]. However, no MHBs-specific antibodies were detected after immunization with these particular VLV, presumably due to interference from the C-terminal 18 amino acids of the T2A peptide, which may have altered MHBs subcellular localization and prevented its secretion [8].

Previously, we evaluated VLV as a vaccine in HBV and HIV preventative models [4,8]. Our HBV studies indicated that VLV elicited HBV-specific immune responses that protected mice from hydrodynamic HBV challenge [8]. Nevertheless, the antigen expression levels and immune responses, although capable of inhibiting HBV replication, were suboptimal and could be further improved. The goal for HBV therapy is to achieve a functional cure, which is characterized by HBV surface (HBs) Ag loss with or without the appearance of anti-HBs antibodies [12]. This can represent a challenge due to peripheral immune tolerance that impedes HBV-specific T cell function [13], which is a consequence of the high serum antigen levels that are present in chronically infected people [14]. Highly immunogenic therapeutic vaccines can potentially break immune tolerance and induce HBV-specific T cells that are functionally able to reduce antigen load and promote the generation of anti-HBs antibodies [8,15]. Most of the past therapeutic vaccine clinical trials evaluated non-replicating platforms, such as proteins or DNA [16,17,18,19,20,21,22], but the use of replicating vaccines presents significant advantages over non-replicating platforms [23,24,25].

Despite the advantages of the VLV platform, the induction of neutralizing antibodies against VSV-G may limit VLV applications to a single immunization or heterologous prime-boost regimens [3,4,8,26]. Switching the envelope glycoproteins in VSV vectors permits homologous prime-boost immunization with VSV and heterologous combinations of VLV and VSV [8,26,27]. In this report, we generated modified VLV constructs that express MHBs and VSV-G using dual subgenomic promoters. We found that dual promoter (dp) VLV possesses significantly increased immunogenicity. We further evaluated the use of Chandipura (CH) virus envelope glycoprotein in VLV vectors and demonstrated that homologous prime-boost immunization with envelope glycoprotein-switched VLV improved MHBs-specific CD8^+^ T cell responses in naïve mice. Moreover, a combination of the VLV dual promoter and glycoprotein switch showed enhanced efficacy in a mouse model of persistent HBV infection.

## 2. Materials and Methods

### 2.1. VLV Dp Vectors

A gene-synthesized (GenScript, Piscataway, NJ, USA) 487-bp DNA fragment containing SFV subgenomic promoter sequence flanked by *Pac*I, *Asc*I and *Sbf*I restriction sites was cloned into the *Bam*HI and *Spe*I sites of the pCMV-SFVG-P50R plasmid [7] to add the second identical subgenomic promoter. VSV Indiana G was cloned into *Bam*HI and *Pac*I restriction sites downstream of the first subgenomic promoter of the resulting vector. HBV MHBs with an added stop codon at the native position (thus removing T2A peptide sequence) and flanking *Asc*I and *Sbf*I cloning sites was amplified from MT2A VLV plasmid DNA [8] using Q5^®^ high-fidelity DNA polymerase and cloned into the *Asc*I and *Sbf*I cloning sites downstream of the first subgenomic promoter. 

To accomplish the envelope glycoprotein switch or change of the MHBs position, DNA fragments encoding MHBs and envelope glycoprotein from Indiana VSV or Chandipura virus were PCR amplified using Pfu proof-reading DNA polymerase (Agilent, Santa Clara, CA, USA) from other VLV vectors or gene-synthesized DNA fragments, and were gel purified. The VLV dp vector was linearized with *Bam*HI/*Pac*I restriction digest to insert the MHBs fragment into the first position and with *Asc*I/*Sbf*I restriction digest to insert envelope glycoproteins into the second position using the NEBuilder^®^ HiFi DNA Assembly kit (New England Biolabs). All selected clones were verified by DNA sequencing at GENEWIZ or the Keck Biotechnology Resource Laboratory at Yale University. Recombinant VSV expressing MHBs from the fifth genome position was previously described [28,29]. 

For production of VLV stocks, BHK-21 cells were obtained from the laboratory of Dr John Rose in the Department of Pathology at Yale University and cultured in Dulbecco’s Modified Eagle Medium supplemented with 5% fetal bovine serum. VLVs were produced by transfection of BHK-21 cells with the VLV plasmid DNA followed by collection of the master VLV stock. Propagation of the working stocks was performed by single passage of the master stock in BHK-21 cells cultured in Opti-MEM™ I Reduced Serum Medium (ThermoFisher, Waltham, MA, USA). Working stocks were concentrated using MacroSep^®^ Advance 100K MWCO (Pall Laboratories, Port Washington, NY, USA) and titrated using plaque assay in BHK-21 cells.

### 2.2. Peptide Epitopes

Overlapping peptide pools and previously described HBsAg CD8^+^ T cell epitopes were used for T cell stimulation, as well as the non-specific HBV peptide controls HBP 140 (HYFQTRHYL) and Core 93 (MGLKFRQL). S 353 is an immunodominant H2-K^b^-restricted HBsAg CD8^+^ T cell epitope consisting of amino acids (a.a.) 353–360 (VWLSVIWM) [30]. S 371 is an immunodominant H2-K^b^-restricted HBsAg CD8^+^ T cell epitope that consists of a.a. 371–378 (ILSPFLPL) [30,31]. Overlapping pools of HBsAg peptides that cover the entire protein sequences (GenScript) or a pool derived from a peptide scan of HBV large envelope protein (HBV-PepMix-L; JPT Peptide Technology) were also used for T cell stimulation.

### 2.3. Immunofluorescence

BHK-21 cells were seeded on pre-cleaned coverslips in 6 well plates at a density of 4 × 10^5^ cells/well for 24 h. After infection with VLVs (MOI = 0.5) for 18 h, cells were washed with phosphate buffered saline (PBS), fixed in 3% paraformaldehyde for 30 min and permeabilized with 0.1% Triton X-100 in 3% FBS/PBS for 15 minutes. Primary (anti-preS2, Santa Cruz) and secondary (anti-mouse AlexaFluor-488, Biolegend, San Diego, CA, USA) antibody staining was carried out in 3% FBS/PBS, followed by extensive washes and counterstaining with 4′,6-diamidino-2-phenylindole (DAPI) (Invitrogen, Waltham, MA, USA). Coverslips were mounted on glass slides with a drop of Prolong^TM^ Diamond antifade mounting medium (Invitrogen). Imaging was performed using a Zeiss LSM 780 combined confocal/FCS/NLO system mounted on an inverted Axio Observer Z1. Diode (wavelengths 405 and 440) and Argon (wavelengths 458, 488 and 514) lasers were used. Zen Black software was used for image acquisition, and Imaris 7.2.3 was used for image analysis (Bitplane, Concord, MA, USA).

### 2.4. Immunoblotting

BHK-21 cells were seeded in 100 mm tissue culture plates one day before infection with VLVs at MOI = 1. Cell lysates were prepared by scraping the cells in cold lysis buffer containing 1% Igepal (Sigma, St. Louis, MO, USA) and Complete^TM^ protease inhibitor cocktail (Roche, Pleasanton, CA, USA) and preclearing by centrifugation at 14,000 *g* for 10 min at 4 °C. Proteins were separated by SDS-PAGE in 4–15% precast gradient gels (Bio-Rad, Hercules, CA, USA) and transferred onto nitrocellulose membranes, which were subsequently blocked and incubated with mouse monoclonal anti-PreS2 (clone S26, Santa Cruz, diluted 1:200), rabbit polyclonal anti-VSV-G (generated in the laboratory of Dr Rose, diluted 1:5000) or anti-actin (clone C4, Millipore, diluted 1:5000) antibodies and HRP-conjugated secondary antibodies (ThermoFisher, diluted 1:5000). A ChemiDoc imaging system (Bio-Rad) was used to acquire and process the images.

### 2.5. Fluorescent Anti-HBs Assay

Recombinant HBsAg (GenScript) was pre-adsorbed overnight in high binding μClear 96-well plates (Greiner, Monroe, NC, USA) at 2 μg/mL in PBS. After 1 h blocking with 3% FBS in PBS, serum samples were diluted in 3% FBS/PBS with 0.1% Tween 20 and incubated for at least 1 h. After washing with 0.1% Tween in PBS, all wells were incubated with donkey anti-mouse polyclonal antibody conjugated with AlexaFluor-680 for 1 h. After washing, the plates were scanned and analyzed with an Odyssey^®^ imaging system (LI-COR Biotechnology, Lincoln, NE, USA).

### 2.6. Immunizations

Naïve C57BL/6 or C57BL/6 mice that were previously transduced with AAV-HBV and were found to have stable antigenemia received 1 × 10^8^ PFU VLV dp intraperitoneally (i.p.) in 200 μL PBS per mouse. The boost was done four weeks after the prime and similarly consisted of 1 × 10^8^ PFU VLV dp i.p. in 200 μL PBS per mouse. Prime-boost immunizations were done by alternating the vesiculovirus glycoprotein expressed by each vector. For VSV immunization, 1 × 10^6^ PFU of virus was administered by the intramuscular route [8].

### 2.7. AAV-HBV Transduction

To establish lower levels of HBV replication, male C57BL/6 mice were transduced with 3 × 10^10^ genome copies of AAV-HBV 1.2-mer (SignaGen, Rockville, MD, USA). In those experiments where more elevated levels of HBV were desired, mice received 1 × 10^11^ genome copies of AAV-HBV 1.2-mer, and animals with intermediate (~500 ng/mL) or higher (~3000 ng/mL) levels of HBsAg were selected for the immunization groups. To assure stable antigen levels, persistent HBV replication was determined by measuring HBsAg levels in the serum at week 8 post-transduction. Animals were placed in experimental groups such that there were no statistically significant differences in antigen levels before immunization.

### 2.8. Isolation of Intrahepatic Leukocytes (IHL)

To obtain IHL, mice were euthanized, the portal vein was cut, and the liver was perfused with PBS. Afterward, the liver was mechanically dissociated and passed through a 100 μM mesh strainer. The cells were purified with 40% Percoll in serum-free media by a 20 min centrifugation at 600× *g* with no brake at room temperature.

### 2.9. Intracellular Cytokine Staining by Flow Cytometry

Detection of HBV-specific interferon (IFN)-γ-producing and IFN-γ/tumor necrosis factor (TNF)-α-producing CD8^+^ T cells was performed by flow cytometry after peptide stimulation of splenocytes or IHL. After cells were collected from the spleen or liver, red blood cells were lysed, and cells were stimulated with peptide for 5 hours in the presence of brefeldin A and monensin. After peptide stimulation, the cells were stained for surface markers (CD8 clone 53-6.7, BioLegend) in the presence of FcBlock (BD Biosciences) followed by intracellular staining for IFN-γ and/or TNF-α (clones XMG1.2 and MP6-XT22 respectively, BioLegend), using a Fixation/Permeabilization Kit (BD Cytofix/Cytoperm™). Data were collected using an LSR II flow cytometer and analyzed with FlowJo software.

### 2.10. Enzyme-Linked Immunosorbent Spot (ELISPOT) Assay

IFN-γ-producing CD8^+^ T cells were measured by IFN-γ ELISPOT assay as previously described [32]. Briefly, spleen cells were harvested, red blood cells were lysed, and 2 × 10^5^ cells were dispensed per well in a plate that had been previously pre-coated with anti-IFN-γ antibody and blocked with complete media. Cells were stimulated overnight at 37 °C with peptides. Plates were rinsed and incubated with secondary biotinylated antibody to IFN-γ for two hours at room temperature, followed by a one-hour incubation with streptavidin-HRP. Finally, spots were developed by incubation with chromogen-substrate solution and were quantified with an automated spot counter (Immunospot, Cellular Technology Ltd, Shaker Heights, OH, USA).

### 2.11. Alanine Aminotransferase (ALT) Measurement

Serum ALT activity was measured using Infinity ALT Liquid Stable Reagent (Thermo Scientific) with Enzyme ER Verifier Kit (Verichem Laboratories, Providence, RI, USA) standards, and analyzed using a SpectraMax iD3 microplate reader (Molecular Devices, San Jose, CA, USA).

### 2.12. Enzyme-Linked Immunosorbent Assay (ELISA) Detection of Viral Antigens and Antibodies

HBsAg, HBeAg, and HBsAb in mouse sera were measured by ELISA (International Immunodiagnostics, Foster City, CA, USA). HBs and HBe Ag standards (Fitzgerald Industries, Acton, MA, USA) were used to quantify Ag levels.

### 2.13. Serum Viral DNA Purification and Detection

To quantify HBV DNA in the serum, viral DNA was purified using a High Pure Viral Nucleic Acid Kit (Roche) following the manufacturer’s instructions. HBV genomes were detected by quantitative PCR using the primers and probe described for HBV RNA analysis below. A plasmid encoding the HBV genome was used as a standard to calculate DNA copy number.

### 2.14. Viral RNA Detection by QPCR

Liver tissue was collected, snap-frozen in liquid nitrogen, and stored at −80^o^ C until processed. RNA extraction and purification, and cDNA preparation were done as previously described [26]. Taqman Fast Advanced Master Mix (Applied Biosystems, Foster City, CA, USA) was used for HBV quantitative PCR. Reactions were done on a StepOnePlus real time PCR system (Applied Biosystems) using StepOne software v2.3. The following primer sequences were used: HBV probe 5’-CCT CTT CAT CCT GCT GCT ATG CCT CAT C-3’, antisense 5’-GAC AAA CGG GCA ACA TAC CTT-3’, sense 5’- GTG TCT GCG GCG TTT TAT CA-3’ [33]. Glyceraldehyde 3-phosphate dehydrogenase (GAPDH) expression was measured as an endogenous control, and gene expression was quantified by the comparative ΔΔC_T_ method.

### 2.15. Statistical Analysis

To determine the difference between the experimental groups, we used 1-way analysis of variance (ANOVA) for endpoint analyses or 2-way ANOVA for analyses over the time course (HBsAg) or between the groups (HBV-specific T cells) with Sidak’s multiple comparison test. For the studies with established HBV replication, paired t-tests were used to analyze HBs and HBe Ag, while unpaired t-tests were done to compare RNA, ELISPOT, and IHL cell percentages. To perform all calculations, we used GraphPad Prism software, versions 7 or 8 (GraphPad Software, San Diego, CA, USA).

## 3. Results

### 3.1. Improved MHBs Expression and Secretion with Double Subgenomic Promoter VLV

**Figure 1:**
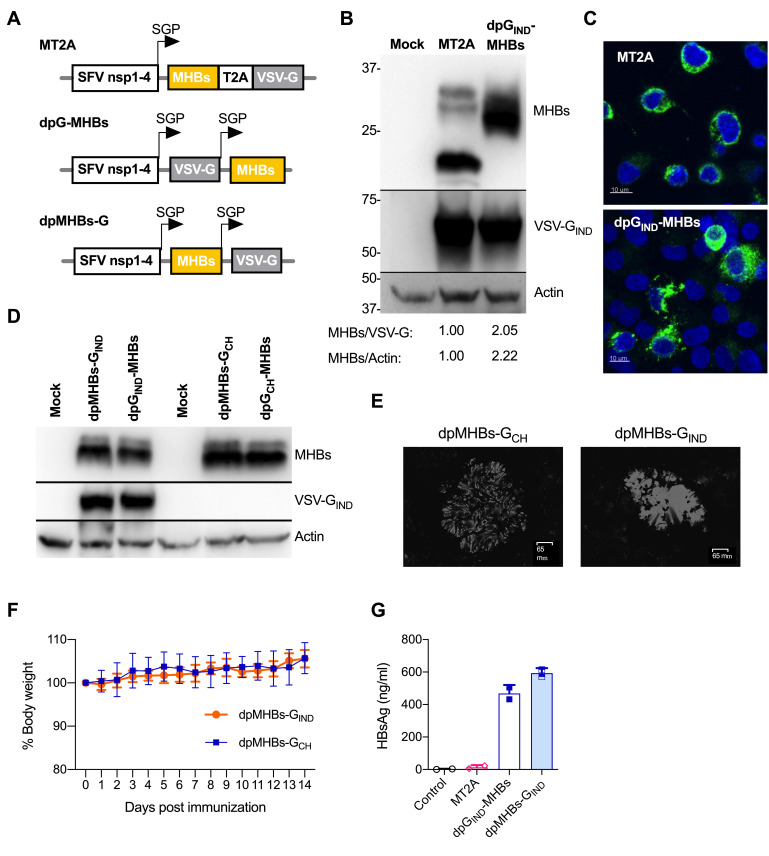
Improved MHBs expression and secretion using VLV dp vectors. (**A**) Schematic of the double subgenomic promoter (SGP) VLV vectors used in this study. (**B**) Immunoblot analyses of MHBs and VSV-G expression in BHK-21 cells at 24 h post-infection with MT2A or dpG_IND_-MHBs VLV (MOI = 1). Densitometry values are for MHBs signal (PreS2) relative to VSV-G or actin. (**C**) Immunofluorescence analyses of MHBs expression (green) in BHK-21 cells at 18 h post-infection with MT2A or dpG_IND_-MHBs VLV at MOI = 0.5. Nuclear counterstaining (DAPI) is shown in blue. (**D**) MHBs expression in BHK-21 cells at 24 h after infection with dp VLV with serotype switch of the envelope glycoprotein from Indiana to Chandipura (IND vs. CH) or position of MHBs (all at MOI = 1). (**E**) Representative plaques from VLV dpMHBs-G_CH_ and VLV dpMHBs-G_IND_ in BHK-21 cells. Plaques were stained with crystal violet and images were obtained using a ZOE fluorescent cell imager (Bio-Rad laboratories). (**F**) Percentage of body weight after intranasal infection with VLV dpMHBs-G_CH_ and VLV dpMHBs-G_IND_. (**G**) Secretion of MHBs measured by ELISA using conditioned media from BHK-21 cells infected with the indicated VLV (all at MOI = 1).

A previously described VLV vector, MT2A, uses a T2A peptide for MHBs expression in frame with VSV-G from a single subgenomic RNA but adds 18 amino acids to the MHBs C-terminus [8], which may cause aberrant processing and secretion of MHBs from infected cells. To determine whether expression of MHBs without any C-terminal modifications improves its production and secretion, we constructed a double subgenomic promoter VLV (VLV dp) vector dpG_IND_-MHBs expressing VSV-G of Indiana strain (IND) and MHBs under separate subgenomic promoters (Figure 1A). Since MHBs in this vector is at the 3’-end of the VLV RNA, we were concerned about the potential loss of MHBs during VLV replication. Therefore, we also designed an alternative vector with MHBs upstream of VSV-G, each expressed from separate subgenomic promoters (dpMHBs-G_IND_) (Figure 1A).

Immunoblot analysis of MHBs in dpG_IND_-MHBs-infected cells showed an increase in protein expression over MT2A and a single band of ~30 kDa instead of several bands in MT2A (Figure 1B), which is likely related to the MHBs glycosylation state [34]. Immunofluorescence analyses of MHBs expression showed a punctate cytoplasmic staining pattern suggesting at least partial co-localization with ER membranes (Figure 1C). Neither changing the position of MHBs in the VLV vector nor switching the envelope glycoprotein from VSV Indiana (G_IND_) to Chandipura virus (G_CH_) had any noticeable effect on MHBs expression as analyzed by immunoblotting (Figure 1D) or VLV titer produced by transfection and single passage (data not shown). As expected, a polyclonal antibody raised by immunization with Indiana vesiculovirus showed no reactivity against G_CH_ (Figure 1D) [27]. VLV dp expressing Chandipura virus glycoprotein formed plaques in BHK-21 cells that are morphologically distinct from those expressing VSV Indiana glycoprotein (Figure 1E), and importantly, no weight loss was observed when mice received intranasal administration of VLV expressing Chandipura virus glycoprotein, similar to VLV expressing Indiana glycoprotein (Figure 1F). Overall, these results demonstrate that Chandipura virus glycoprotein, which is serologically distinct from VSV Indiana glycoprotein, is competent for enabling VLV replication with in vitro evolved SFV nsp1–4.

To determine whether MHBs was secreted when expressed by the VLV dp vector, we analyzed HBs in conditioned media from BHK-21 cells infected with MT2A, dpG_IND_-MHBs, or dpMHBs-G_IND_ for 24 h (MOI = 1) by ELISA. MHBs levels after infection with MT2A were consistently below 25 ng/mL, just two-fold above the limit of detection (12 ng/mL). However, MHBs levels were present in the media at concentrations of 400–600 ng/mL after infection with either dpG_IND_-MHBs or dpMHBs-G_IND_ (Figure 1G), indicating greater MHBs secretion. Importantly, no cytopathic effect was observed under these conditions, ruling out passive MHBs release from infected dying cells (data not shown).

### 3.2. VLV Dp Generate Enhanced CD8^+^ T Cell and Antibody Responses

**Figure 2:**
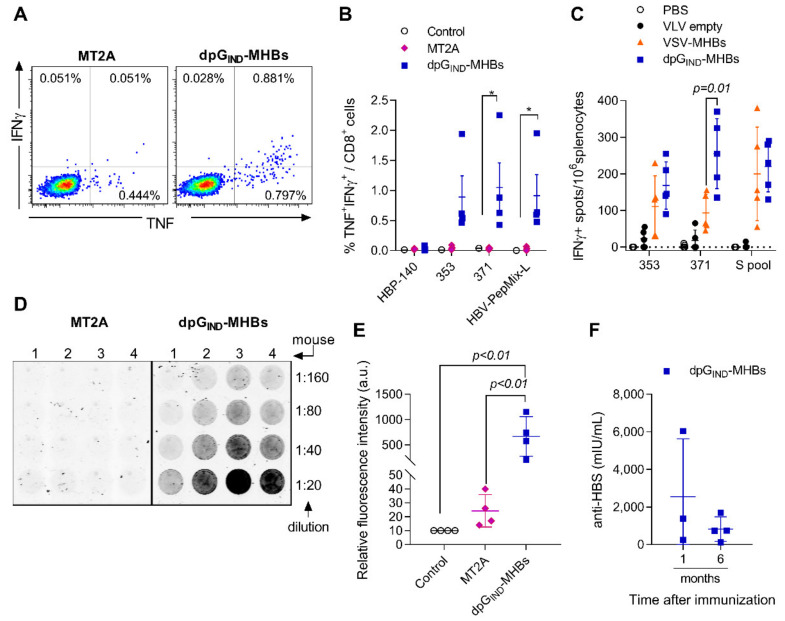
MHBs expression with dp VLV enhances MHBs immunogenicity in naïve mice. C57BL/6 male mice were immunized with 1 × 10^8^ PFU/mouse MT2A or dpG_IND_-MHBs to analyze splenic effector T cell and antibody responses. (**A**) Representative dot plots showing intracellular staining for TNF and IFN-γ in gated CD8^+^ T cells after re-stimulation with HBS-371 peptide. (**B**) Frequency of TNF^+^IFNγ^+^ cells among CD8^+^ T cells at day 7 post-immunization measured by intracellular staining and flow cytometry after re-stimulation with the indicated peptides or peptide pool. (**C**) Frequency of IFNγ+ cells measured by ELISPOT after re-stimulation with the indicated peptides or peptide pool at day 14 post-immunization with empty vector VLV, dpG_IND_-MHBs, or VSV-MHBs. VSV-MHBs was used at 1 × 10^6^ PFU/mouse by the i.m. route of immunization. (**D**) Detection of anti-HBs antibodies using solid-phase absorbed HBsAg. Dilutions of mouse serum samples collected at 1 month after immunization and fluorescently conjugated anti-mouse secondary antibody. (**E**) Quantification of the signal from the anti-HBs assay at the 1:20 dilution of the serum samples. (**F**) Measurements of anti-HBs antibodies at 1 month and 6 months post-immunization with dpG_IND_-MHBs. * *p* < 0.05, relative to control group.

Since dual promoter VLV constructs showed higher MHBs expression and secretion, we compared the immunogenicity of the dpG_IND_-MHBs and MT2A VLV in naïve mice after a single immunization. Intracellular cytokine staining followed by flow cytometry analysis showed a statistically significant increase in MHBs-specific IFNγ^+^TNF^+^ CD8^+^ T cell frequency in the group of mice immunized with dpG_IND_-MHBs compared to the MT2A-immunized group (Figure 2A,B). The increase in HBV-specific CD8^+^ T cells was evident after stimulation with individual peptides from the HBV surface antigen (353 and 371) or peptide library (HBV-PepMix-L), but not with a non-specific control peptide, HBP-140. To further evaluate the overall immunogenicity of VLV dp vectors, we immunized naïve mice with either VSV or VLV dp expressing MHBs. Previously, we found VSV expressing MHBs to be highly immunogenic [28,29], so comparing the two vectors can help define VLV dp immunogenicity. After immunization, we measured the generation of IFN-γ-producing MHBs-specific CD8^+^ T cells, and found that VLV dpG-MHBs and VSV-MHBs have a similar capacity to generate MHBs-specific CD8^+^ T cells (Figure 2C).

In previous studies, MT2A VLV immunization failed to induce anti-HBs antibodies [8]. To increase the sensitivity of anti-HBs detection, we customized an immunosorbent assay by using solid-phase absorbed HBsAg and fluorescent-conjugated anti-mouse secondary antibody. Serum samples collected at 4 weeks after immunization with MT2A showed a signal barely above background at 1:20 dilution (Figure 2D,E). However, serum samples from a dpG_IND_-MHBs-immunized group showed a detectable signal at dilutions of 1:20 to 1:160. Using a commercially available quantitative ELISA, we found that 3 out of 4 animals immunized with dpG_IND_-MHBs showed anti-HBs antibody levels above background (ranging from 243 to 6040 mIU/mL) at 1 month after immunization (Figure 2F). Six months after the single immunization, all the animals immunized with dpG_IND_-MHBs had anti-HBs antibody levels above background (ranging from 118 to 691 mIU/mL). Together, these data demonstrate that immunization with dpG_IND_-MHBs induced anti-HBs antibodies in naïve mice.

### 3.3. Homologous Prime-Boost Immunization with Envelope Glycoprotein Switch Enhances the Magnitude of MHBs-Specific CD8^+^ T Cell Responses

**Figure 3:**
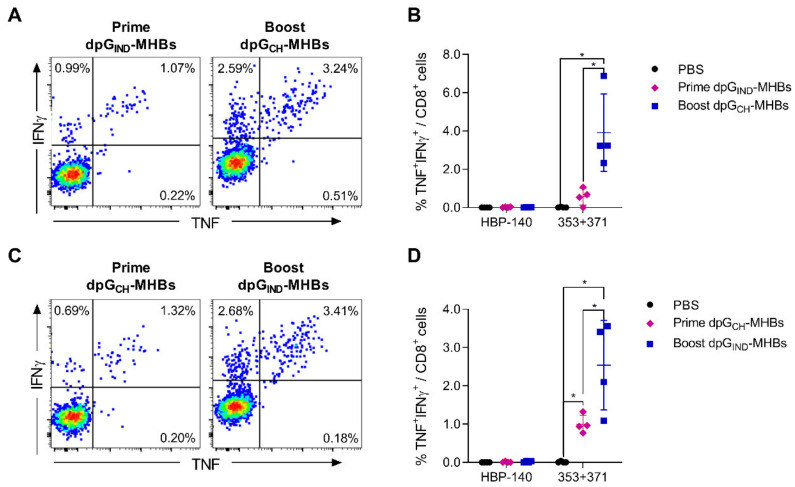
VLV dp homologous prime-boost immunization with glycoprotein switch enhances MHBs-specific splenic CD8^+^ T cell responses. C57BL/6 male mice were immunized with 1 × 10^8^ PFU/mouse dpG_IND_-MHBs or dpG_CH_-MHBs. A subset of the mice (*n* = 4 in each group) was euthanized to measure effector responses at day 7 post-immunization (prime). The remaining mice (*n* = 4) were boosted with VLV having the opposite glycoprotein serotype at 4 weeks after the prime and euthanized 5 days later to measure the effect of the boost. Representative dot plots showing intracellular staining for TNF and IFN-γ in gated CD8^+^ T cells after re-stimulation with pooled 353 and 371 peptides are shown in (**A**) and (**C**). Frequency of the MHBs-specific CD8^+^ T cells (TNF^+^IFNγ^+^) is shown in (**B**) and (**D**). * *p* < 0.05, relative to PBS or prime groups, as indicated.

The ability to boost with virus vectors is often constrained by pre-existing immunity or de novo induction of neutralizing antibodies against the vaccine platform proteins. We anticipated that VLV vectors would have the same limitation since VSV-G, the sole viral structural protein present on the VLV surface, is highly immunogenic for neutralizing antibody production [35,36]. Previous studies found that exchanging the VSV-G protein serotype can prevent neutralization when performing prime-boost with VSV [27]. We used this technique to exchange the VSV Indiana serotype for Chandipura virus in a prime-boost immunization regimen of dpG-MHBs VLV. Under these conditions, the HBV MHBs-specific CD8^+^ T cell responses were boosted about 3-fold (Figure 3A,B). Switching the glycoprotein order, i.e., priming with VLV dp Chandipura and boosting with VLV dp Indiana, resulted in similar HBV-specific CD8^+^ T cell frequency enhancement (Figure 3C,D).

### 3.4. A Single Dose of VLV Dp Controls Established HBV Replication in Mice with Lower Antigenemia in the AAV-HBV Model

**Figure 4:**
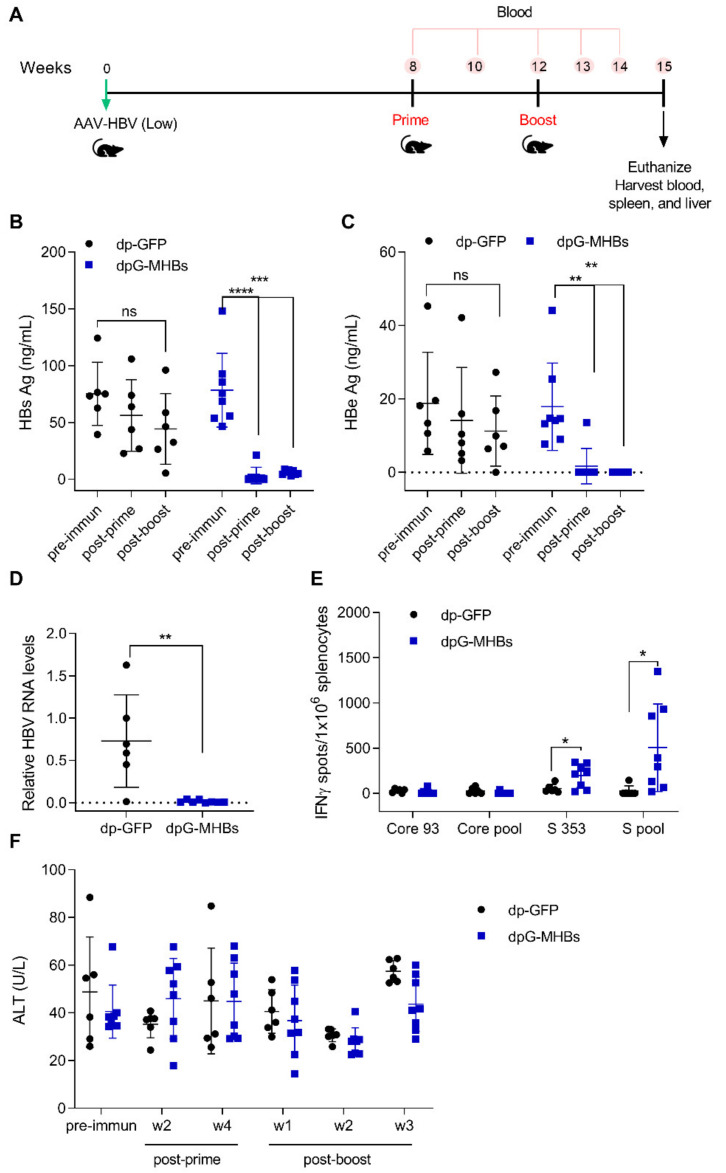
VLV dp immunization controls HBV replication in mice with pre-existing lower HBV antigen levels. (**A**) Mice were transduced with 3 × 10^10^ genome copies of AAV-HBV and after HBV replication was established received a prime-boost immunization with VLV dp. HBV antigen levels, liver RNA, and T cell responses were analyzed following vaccination. Serum (**B**) HBsAg and (**C**) HBeAg were measured at different time points pre- and post-immunization (two weeks post-prime and three weeks post-boost). (**D**) Liver RNA was quantified by RT-qPCR with GAPDH as the endogenous control 3 weeks after the final boost. (**E**) IFN-γ-producing cells in the spleen of immunized mice after stimulation with Core and S antigen peptides. (**F**) Serum ALT levels in immunized mice during the time course of the experiment. * *p* < 0.05, ** *p* < 0.01, *** *p* < 0.001 relative to dp-GFP or the pre-immunization time point.

To evaluate the ability of VLV dp homologous prime-boost to eliminate established HBV replication, mice were transduced with AAV-HBV and subsequently immunized as shown in Figure 4A. Mice that received VLV dpG-MHBs showed a reduction of HBs and HBe Ag as soon as two weeks after the prime (Figure 4B,C), which persisted over time to levels below the limit of detection 3 weeks after a boost. In contrast, those mice that received the non-specific VLV dp-GFP did not clear HBs and HBe, despite a small reduction in Ag levels (Figure 4B,C). Consistent with the reduction of antigen levels, mice that received VLV dpG-MHBs had no detectable HBV RNA in the liver at week 3 post-boost, in contrast to mice that received VLV dp-GFP (Figure 4D).

Since VLV dp prime-boost elicits HBV-specific CD8^+^ T cell responses in naïve mice (Figure 3), we evaluated whether VLV dp prime-boost also generated HBV-specific responses in mice with pre-established HBV replication. At 3 weeks after the boost, only mice immunized with VLV dpG-MHBs had significant MHBs-specific IFN-γ-producing CD8^+^ T cells in the spleen (Figure 4E). Despite the presence of these CD8^+^ T cell responses and control of HBV in the immunized mice, no significant changes in the ALT levels were observed (Figure 4F). This could be explained by the initial low HBV antigen levels, which may not evoke significant liver damage upon HBV elimination. Additionally, we measured antibody levels induced with VLV dp and found that only 3 out of 8 mice had detectable titers of anti-HBs antibody (data not shown), further suggesting that HBV elimination is primarily CD8^+^ T cell-mediated.

### 3.5. Prime-Boost with VLV Dp Reduces HBV Levels in Mice with Pre-Existing Intermediate Ag Levels

**Figure 5:**
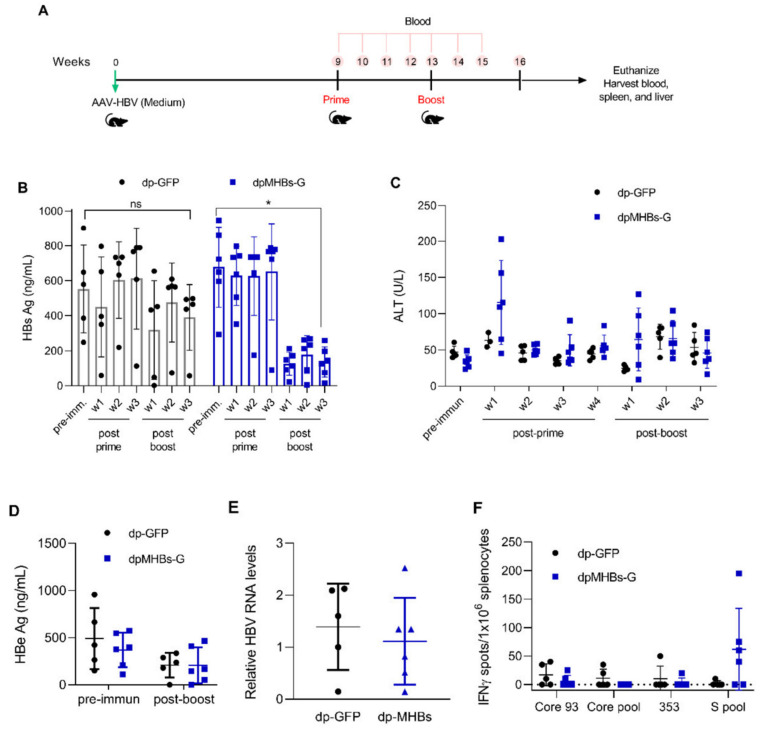

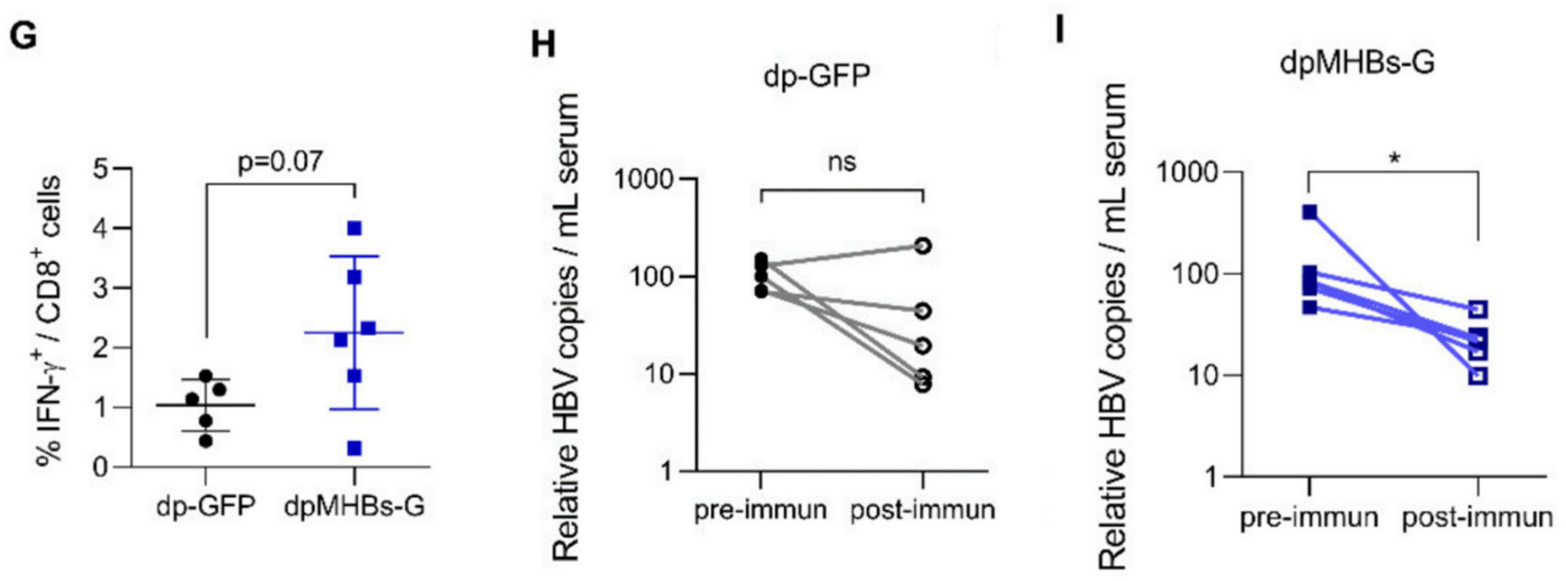
VLV dp prime-boost immunization significantly reduces HBV in mice with pre-existing intermediate HBV antigen levels. (**A**) Mice received 1 × 10^11^ genome copies of AAV-HBV followed by VLV prime-boost immunization. Analyses of T cell responses and antigenemia were then performed. (**B**) Serum HBsAg levels in the mice at the indicated time points pre- and post-immunization. (**C**) Serum ALT pre- and post-immunization. (**D**) Serum HBeAg levels in the mice pre- and post-immunization. (**E**) Liver RNA was quantified by RT-qPCR with GAPDH as the endogenous control 3 weeks after the final boost. (**F**) IFN-γ-producing cells in the spleen of immunized mice after stimulation with Core and S antigen peptides. (**G**) Percentage of IFN-γ-producing cells in the IHL of immunized mice after stimulation with S antigen peptides. (**H**,**I**) Serum HBV DNA pre- and post-immunization with (**H**) dp-GFP and (**I**) dpMHBs-G. * *p* < 0.05, ns = not significant compared to pre-immunization.

Since a single immunization efficiently controlled HBV replication in mice with lower HBV Ag levels (<100 ng HBsAg/mL), we reasoned that prime-boost immunization might control HBV in mice with more elevated antigen levels (average HBsAg ~500 ng/mL). We therefore transduced mice with a higher AAV-HBV dose, screened for animals with the desired antigen levels, and immunized with VLV dp as shown in Figure 5A. In contrast to mice with lower Ag levels, a single VLV dp dose did not decrease HBsAg levels (Figure 5B). Nevertheless, mice that received a VLV dpMHBs-G prime-boost had significantly reduced HBsAg (Figure 5B). Only those mice that received VLV dpMHBs-G showed transient ALT elevation (Figure 5C) that is consistent with HBV-specific CD8^+^ T cell responses in the spleen (Figure 5F) and liver (Figure 5G) of the immunized mice. In these mice however, no significant changes in serum HBeAg (Figure 5D) or HBV liver RNA (Figure 5E) were observed, although there was a significant reduction of serum HBV DNA in mice immunized with VLV dpMHBs-G but not VLV dp-GFP (Figure 5H,I).

### 3.6. VLV Dp Prime-Boost Immunization Does Not Control HBV in Mice with Pre-Existing Higher HBV Antigen Levels

**Figure 6:**
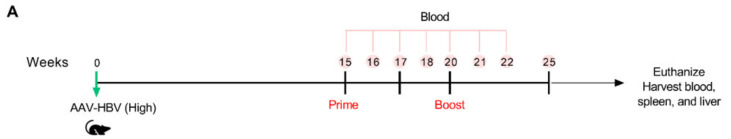

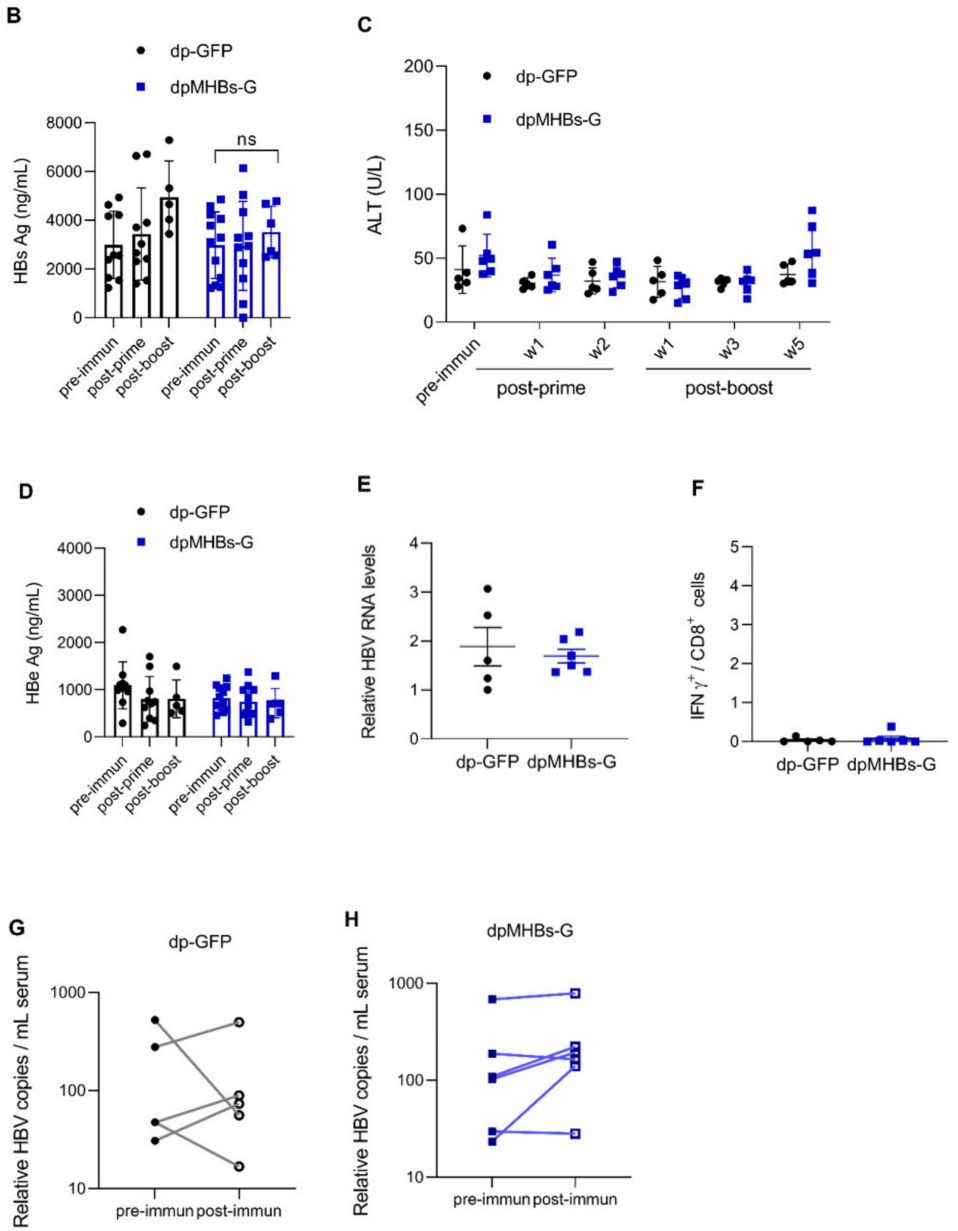
VLV dp prime-boost immunization does not control HBV in mice with pre-existing higher HBV antigen levels. (**A**) Mice received 1 × 10^11^ genome copies of AAV-HBV followed by VLV prime-boost immunization. Analyses of T cell responses and antigenemia were then performed. (**B**) Serum HBsAg levels in the mice pre- and post-immunization. (**C**) Serum ALT pre- and post-immunization. (**D**) Serum HBeAg levels in the mice pre- and post-immunization. (**E**) Liver HBV RNA was quantified by qPCR with GAPDH as the endogenous control 5 weeks after the final boost. (**F**) Percentage of IFN-γ-producing cells in the IHL of immunized mice after stimulation with S antigen peptides. (**G**,**H**) Serum HBV DNA pre- and post-immunization with (**G**) dp-GFP and (**H**) dpMHBs-G. ns = not significant compared to pre-immunization.

Prime-boost immunization in mice with intermediate HBV Ag levels had a moderate impact on HBV control. To further understand the effect of antigenemia on VLV dp-mediated HBV control, we evaluated anti-HBV efficacy in mice with even higher antigen levels. We transduced mice with AAV-HBV and screened for elevated HBs antigenemia (average HBsAg ~3000 ng/mL), followed by immunization with VLV dp as shown in Figure 6A. In contrast to mice with lower and intermediate Ag levels, mice that received VLV dpMHBs-G prime-boost did not show decreased serum HBsAg levels (Figure 6B). Furthermore, there were no changes in serum ALT (Figure 6C), HBeAg (Figure 6D), liver HBV RNA (Figure 6E), IHL IFN-γ production (Figure 6F), or serum HBV DNA (Figure 6G,H). Together, these data suggest that HBV Ag levels influence the ability of VLV dp prime-boost immunization to control HBV replication.

## 4. Discussion

In this study, we produced a modified dual promoter VLV and found that dpG-MHBs was highly effective in reducing serum HBsAg and liver HBV RNA below the lower limit of detection in the AAV-HBV mouse model of chronic hepatitis B when mice had initial lower HBV antigen levels (Figure 4). There was also a temporary reduction in HBsAg two weeks following immunization in the VLV dp-GFP control group (Figure 4B), which might be explained by activation of the innate immune response by the VLV platform. Although the control VLV alone is not sufficient to clear serum HBsAg, the combination of innate and HBV-specific cellular responses that occur with HBV antigen expression from VLV dp was sufficient for clearance. However, when mice with intermediate antigen levels (~500 ng/mL) received a VLV dp homologous prime-boost, significant antigen reduction was observed (Figure 5B) and MHBs-specific CD8^+^ T cell responses could be detected in the liver, although virus reduction was not complete. Despite partial reduction of HBsAg in the serum, no changes in liver HBV RNA were observed 3 weeks after the final boost. This difference could be due to detection of RNA fragments by RT-qPCR, altered ratios of HBV 3.5 and 2.1 kb mRNAs from cytokine-mediated down-regulation of transcription, or low non-saturating levels of anti-HBs antibody not detected by ELISA. When antigen levels were even greater (~3000 ng/mL), there was no reduction in antigenemia, nor detectable MHBs-specific CD8^+^ T cell responses (Figure 6). A second boost with VLV dp could potentially promote further HBV clearance, either alone or in combination with other treatments.

Similar to our previous studies with VLV MT2A [8], we detected MHBs-specific CD8^+^ T cells after a single dose of VLV dp (Figure 2A). Consistent with previous findings, the magnitude of the MT2A-induced responses was low; nonetheless, these responses are functional and can expand after HBV hydrodynamic challenge [8]. Importantly, the modified VLV dp vector generates responses that are significantly greater in magnitude than M2TA (Figure 2A–C and [8]). Furthermore, VLV dp show similar immunogenicity compared to VSV, which indicates that VLV dp are efficient inducers of antigen-specific CD8^+^ T cells.

Anti-HBs antibody production may be essential to attain HBV functional cure [12]. We found that immunization with VLV dpG-MHBs induced strong antibody production in naïve mice (Figure 2D). In contrast, we saw limited production of antibodies when mice with established HBV replication received VLV dp immunizations. This partial antibody response could be explained by a highly tolerant or exhausted environment that prevents antibody generation. If this is the case, additional CD4^+^ T cell help might be necessary to promote adequate antibody responses. This may be achieved through inclusion of additional HBV antigens, such as core and/or polymerase, combination with antibodies to stimulate immune checkpoint blockade [37,38,39] or to agonize other immunostimulatory receptors such as 4-1BB or OX40 [40,41,42].

An essential feature of this modified VLV dp vector is the addition of a second subgenomic promoter. There are two possible consequences of using this promoter to drive MHBs expression independent of other protein-coding sequences. The first is a higher MHBs expression level, which is observed in the comparison between MT2A (where MHBs is expressed within the MHBs-T2A-G ORF) and dpG-MHBs (where MHBs is expressed independently of VSV-G) (Figure 1B). Second, in the context of dpG-MHBs, MHBs folding may be improved since there are no amino acids added to its C-terminal end, as when MHBs is expressed from MT2A [8]. Expression that is independent of T2A may allow MHBs to proceed through the secretory pathway and be properly embedded within membranes and allow proper glycosylation [43]. Whether this results in MHBs incorporation into the membranes of VLV remains unknown, but regardless of whether the protein is in VLV membranes, the VLV dual promoter vector drives improved immunogenicity for MHBs (Figure 2). Although the present study investigated VLV dp efficacy in a chronic HBV model where T cells are most relevant for virus control, the capacity of VLV dp to generate better antigen-specific antibodies responses compared to the polycistronic construct VLV 3xT2A [10] indicates the potential of VLV dp as a vaccine platform to induce better antibody responses to secreted antigens.

An important component of the VLV platform is VSV-G, which is also a potent immunogen that stimulates production of a neutralizing antibody response. This antibody response restricts the ability to boost after priming with VSV or VLV. However, VSV has several serotypes that are sufficiently different from each other and are not cross-neutralized by antibodies to another serotype [27]. Therefore, using expression of different vesiculovirus glycoproteins on VLVs circumvents this limitation, thus allowing for a substantial boost to the primed immune response initially generated to the encoded antigens (Figure 3). This may be important in the context of the high level of tolerance associated with chronic HBV infection, where a single administration is likely to prove insufficient and boosted responses will likely be required to break tolerance and activate de novo or existing T cell responses to HBV antigens. When the originally engineered VLV were tested using other glycoproteins besides VSV-G, we found that only VSV-G could be recovered with high efficiency and the ability to further propagate [1]. Due to the significant differences between Chandipura virus and VSV Indiana [44], it was imperative to evaluate the competence of VLV dp using Chandipura G to replicate and express foreign antigens. Although we evaluated prime-boost with the Indiana and Chandipura glycoproteins in the context of the dp VLV platform, this approach is likely to improve the immunogenicity of the original single promoter platform as well. The ability to prime and boost with this platform also allows for the potential vaccination against different diseases using VLV, since switching VSV-G serotypes would avoid pre-existing immunity to the platform itself.

## 5. Conclusions

We developed a modified VLV platform that not only showed increased immunogenicity and efficacy in a chronic HBV model, but also has improved features that will allow further expansion of vaccination targets. The ability to prime and boost can be important for other applications, such as vaccination with weakly immunogenic antigens or in situations when long-term immunity is desired. As prime-boost with VLV dp had no detectable effect on HBV replication when antigen levels were high, HBV functional cure will likely require a combination of approaches [12]. Given the observations discussed here, VLV-based therapeutic vaccines may represent an advantageous strategy that in combination with nucleos(t)ide analogs, immune stimulators, or other investigational drugs will reduce antigen levels and promote the generation of functional HBV-specific CD8^+^ T cell responses that ultimately control HBV.
